# Foreign Body Misdiagnosed as Mucus Plugging After Percutaneous Tracheostomy

**DOI:** 10.7759/cureus.49147

**Published:** 2023-11-20

**Authors:** Bothayna Amien, Amer Harky, Amy Hill, Neeraj Mediratta

**Affiliations:** 1 Cardiothoracic Surgery, Liverpool Heart and Chest Hospital, Liverpool, GBR; 2 Anaesthesia and Critical Care, Liverpool Heart and Chest Hospital, Liverpool, GBR

**Keywords:** misdiagnosis, persistent cough, mucus plug, airway foreign body, percutaneous tracheostomy

## Abstract

We report a case of a 59-year-old male who presented with a persistent cough for a year after being discharged from critical care following a subarachnoid haemorrhage. As part of his initial critical care management and in order to allow full neurological assessment, the patient required a period of prolonged mechanical ventilation, which necessitated a percutaneous tracheostomy. Following recovery and subsequent discharge, the patient presented on multiple occasions with cough, undergoing serial computed tomography (CT) scans which reported mucus plugging as a possible cause of the cough. As his symptoms continued to worsen, a flexible bronchoscopy was carried out, which identified a foreign body in the trachea. This object was later recognised as a retained part of the guiding catheter, part of the percutaneous tracheostomy tube dilator. After the object was retrieved, the patient reported a complete resolution of symptoms. Percutaneous tracheostomy is a common procedure within critical care units, and early complications such as bleeding or airway obstruction are typically recognised immediately after insertion. This report documents a late complication caused by the retention of a foreign object from insertion, which was misdiagnosed on serial CT scans, leading to persistent cough over a period of months.

## Introduction

Percutaneous tracheostomy is a common procedure within critical care units, being performed frequently in patients requiring longer-term mechanical ventilation [1]. Lessening the requirement for any sedative agents that may be needed for endotracheal tube tolerance, it can allow for accurate neurological assessment, easier patient communication, and consistent engagement with physiotherapy, among other reported benefits [1,2]. Like all invasive procedures, there is the potential for risk, which must be balanced along with the perceived benefits. Reported major early complications include airway obstruction or bleeding at the insertion site present at the time of insertion with defined and acute signs [3]. Other less life-threatening complications such as tracheostomy site infection are easily identifiable but again follow a more defined and easily diagnosed clinical course [3]. Later complications may present with vague symptoms, leading to delays in diagnosis or misdiagnosis, as demonstrated in this case where a foreign body was misdiagnosed as mucus plugging despite serial CT scans and hospital attendances. 

## Case presentation

The patient, 59-year-old male, had suffered a subarachnoid haemorrhage (SAH) in July 2020, following which he was managed in a neurological critical care unit for a period of two months. His past medical history consisted of a cerebral vascular accident (CVA) in 2015, gastro-oesophageal reflux, and a high BMI of 44 kg/m^2^. He had never smoked. During this admission, he required a period of prolonged ventilation, and a subsequent percutaneous tracheostomy in order to allow for sedation-free neurological assessment and weaning from ventilatory support. After successful tracheostomy decannulation and discharge from the speech and language team allowing him to take an oral diet, he was transferred to a neurological rehabilitation unit due to his persistent right-sided weakness and expressive dysphasia.

After discharge from the rehabilitation unit, he started to report a persistent cough. In March 2021, he was coincidentally admitted to the hospital with *Enterococcus faecalis *septicaemia. At this time, his persistent cough was investigated by CT scan, which reported collapse and consolidation in the right lower lobe (unchanged since 2020), and mild mucus secretions in the trachea. The Ear, Nose, and Throat (ENT) team also reviewed him, and the laryngeal assessment showed right vocal cord weakness, attributed to his previous CVA. 

**Figure 1:**
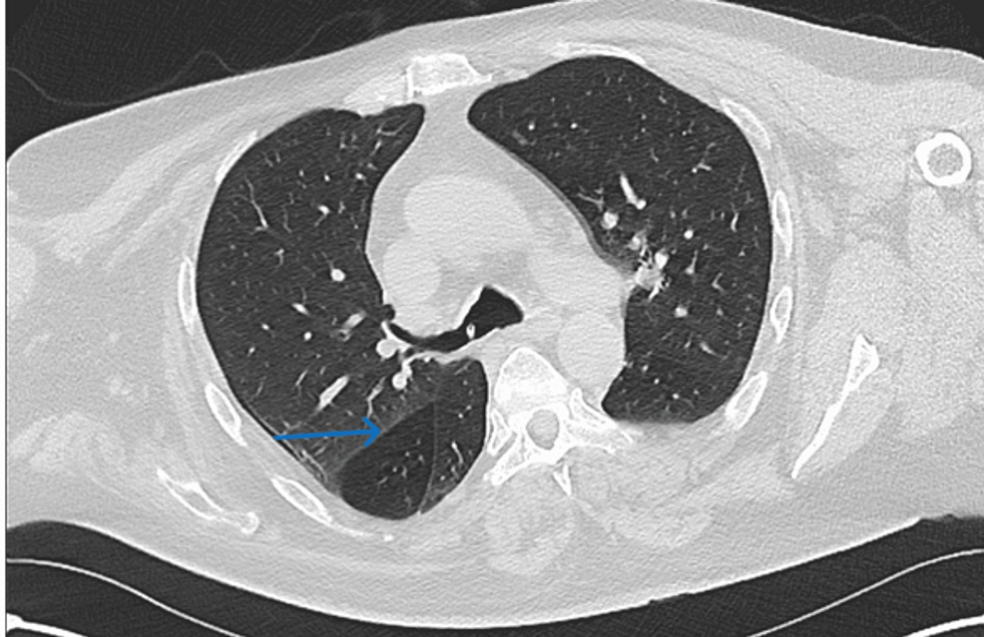
CT scan showing stable appearance of the trachea and right middle and lower lobe bronchi. The partial collapse of the right lobe was attributed to mucus plugging

Upon review by the respiratory team, the symptoms had improved; therefore, further investigation by bronchoscopy wasn’t considered necessary at that time. A repeat CT scan in December 2021 showed a stable appearance of the trachea and right middle and lower lobe bronchi. The partial collapse of the right lobe was attributed to mucus plugging, although radiologists were unable to exclude any endobronchial lesion on imaging completely (Figure 1).

**Figure 2:**
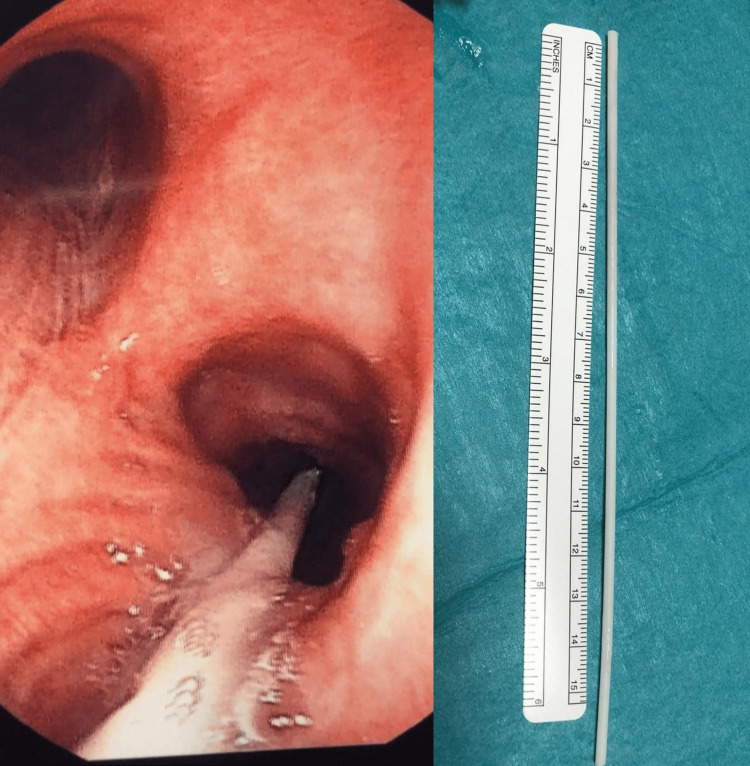
White fine tube-like structure in the trachea reaching the right lower lobe bronchus

When the patient's symptoms recurred after this, a bronchoscopy was performed by the respiratory team who found a white tube-like foreign object, which measured 15 cm and reached the right lower lobe bronchus, in the upper trachea seated between the vocal cords and the carina. The patient was referred to our cardiothoracic unit for assessment and removal of the foreign body that was seated between the vocal cords. The cardiothoracic team retrieved the foreign body using a rigid bronchoscopy under general anaesthesia (Figure 2). 

## Discussion

Tracheostomy is more commonly performed in the critical care setting via the percutaneous method, so as to allow mechanical ventilation along with sedation-free neurological assessment [1]. As an invasive procedure, tracheostomies pose potential risks, and complications can be categorised as early or delayed. In-hospital mortality following tracheostomy has been reported as up to 22% in one study [3]. A systematic review by Delaney et al. looking into tracheostomy complications reported an early (within 48 hours) bleeding incidence of 5.7%, commonly attributed to damage to superficial veins [1]. After 48 hours, any bleeding was more likely to be caused by major vessel involvement, e.g. innominate artery. Stomal infections occur in 6.6% of cases [1,4]. Late complications happen in a further 67% and include delayed closure of the stoma, airway obstruction, tracheal stenosis, tracheomalacia, and tracheoesophageal and tracheoinnominate fistulas [1,3].

The incidence of tracheostomy obstruction has been reported at 0-3.5% [1]. Common causes include mucus plugging and obstruction resulting from clotted blood in the tube. Although this obstruction typically causes a presentation early after tracheostomy insertion, there have been reports of partial obstruction causing chronic symptoms [5]. Miyata et al. reported a case of an 81-year-old woman with a history of dyspnoea and productive cough, having had a double cannula tracheostomy sited 12 years previously. On this occasion, CT diagnosed a foreign body in the trachea thought to be a granuloma but further investigations revealed this to be a mucus plug [5]. 

There are very few documented cases in the literature of retention of a foreign body following tracheostomy, either via the surgical or percutaneous method. The first case report identified dates back to 1960 and reports tracheostomy cannula fragments in the right main bronchus, discovered after presenting with respiratory infection with the tracheostomy tube in place This was attributed to season cracking of the cannula, caused by long-continued high internal stress [6]. Mahattanasakul et al. presented a case series of four patients in whom fractured metallic tracheostomy tubes were identified, with the most common presentations being dyspnoea, intolerable cough, and decreased breath sounds on auscultation [7]. The most severe presentation described was that of one patient who suffered a cardiac arrest. The tracheostomy tubes had been in place for an average number of 24 days, and the dislodged tubes were retrieved by either flexible or rigid bronchoscopy [7]. Another case report described a situation where a polyvinyl chloride (PVC) tracheostomy tube fractured and migrated to the right main bronchus and was retrieved using bronchoscopy [8].

In the above cases, all patients presented with respiratory symptoms. In most, the tracheostomy tube was still in place, making the diagnosis easier. In our case, the patient presented with mild respiratory symptoms over a prolonged period after decannulation, making it difficult to correlate with the previous episode of tracheostomy. Moreover, the foreign body was diagnosed as a mucus plug partly as it fits the history of immobility and stroke. This patient eventually underwent bronchoscopy because of persistent symptoms and the retained foreign body tube then became evident and was retrieved under general anesthesia using rigid bronchoscopy. To the authors' knowledge, this is the first case of a part of the insertion kit of the tracheostomy tube to be dislodged into the trachea.

Mechanism of retained foreign body 

In this report, we describe a case of a foreign body inadvertently left within the airways after the insertion of a percutaneous tracheostomy tube. The usual method of insertion is described below, providing an explanation of why the ‘guiding catheter’ may have been unaccounted for.

**Figure 3:**
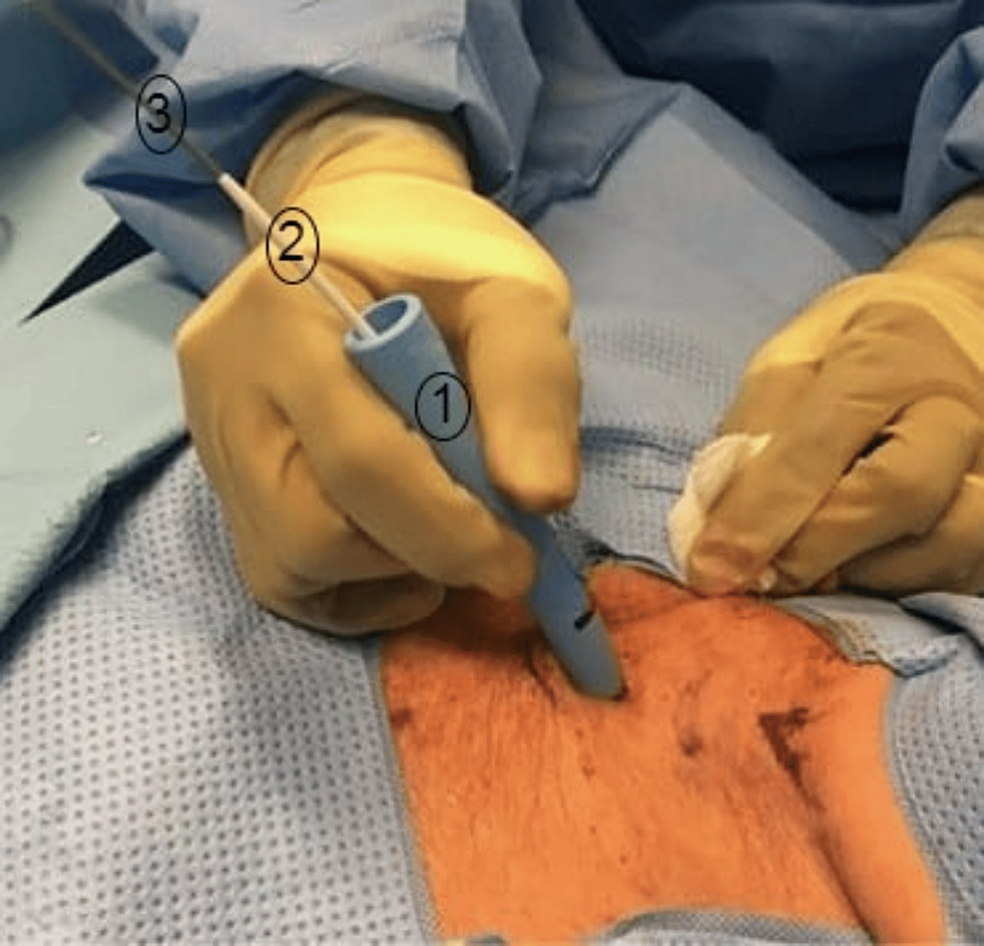
The percutaneous tube dilator (1), guiding catheter (2), and guiding wire (3) Image credit: Rashid and Islam, 2017 [9]; CC BY-NC-ND 4.0 Deed/Attribution-NonCommercial-NoDerivs 4.0 International

Via a Seldinger technique, the introducer needle is first passed into the trachea under bronchoscopic guidance, usually between the first and second or the second and third tracheal rings, aiming for as close to the 12-o clock position as possible. A guidewire is then passed through this needle and observed within the airway again using bronchoscopy. A small incision is usually made to make the passage of dilators easier but is not always required using the classical Seldinger technique (Figure 3) [9]. 

This specific set requires initial dilation with a 14 French ‘introducer dilator’, followed by formal dilation using the larger tracheostomy tube dilator. To smooth the passage of the second, larger dilator’ and avoid trauma to the airways that could be created from the end of the multi-dilator, a narrow and longer ‘guiding catheter’ is placed within the larger dilator, through which the guidewire can pass. The two together provide a smooth and gradual increase in size during dilation over the guidewire and are advanced together within the trachea to the necessary marking. The guidewire is then left within the trachea, and the ‘guiding catheter’ is pulled back in order to allow placement of the definitive tracheostomy over this, again providing a smooth and gradual increase in the size of the kit and avoiding a definitive lip and potential trauma. The tracheostomy, guidewire and ‘guiding catheter’ are then advanced into the trachea, with the ‘guiding catheter’ and guidewire being removed. The final position of the tracheostomy is determined again by bronchoscopy [9].

We propose that the ‘guiding catheter’ was not removed alongside the guidewire after the tracheostomy insertion, most likely due to the need to use this piece of equipment twice, and the requirement to manipulate it to align with both the larger (second) dilator and the actual tracheostomy. In addition to this, components of Seldinger sets are not commonly counted in the same way as surgical swabs and instruments, especially within ward and critical care environments. 

## Conclusions

This case report describes persistent and late symptoms years after a percutaneous tracheostomy. The mild symptoms were initially attributed to mucus plugging but the persistence of symptoms led to the late recognition of the retained part of the guiding catheter from the percutaneous tracheostomy insertion kit. Complications of tracheostomy insertion can occur long after the patient has been decannulated and discharged from the hospital. They may present with mild respiratory symptoms rather than acute airway obstruction, and a high index of suspicion is required in patients with a history of previous tracheostomy who present with ongoing respiratory symptoms. 

We suggest in the event of non-resolving, late symptoms after decannulation, a bronchoscopy should be considered to rule out any retained objects. We also described the mechanism of the retained foreign body; this is to further shed light on a rare incident. This case emphasises the need for careful counting of all parts of Seldinger procedure kits, in order to prevent similar occurrences in the future. 
